# Asiatic Cholera

**Published:** 1866-12

**Authors:** 


*Asiatic Cholera.—A treatise on its origin, pathology, treatment, and cure. By E. Whiting, M. D., and A. B. Whiting, M. D.—M. W. Dodd, Publisher, New York.*

This is a small volume of 212 pages, neatly printed and bound. Time has not allowed us sufficient perusal of its contents to form an opinion of the full merits of the work. We will take occasion hereafter to allude to it.

